# Assessment of Cognition in Hypertensives and Normotensives: A Comparative P300 Study

**DOI:** 10.7759/cureus.28397

**Published:** 2022-08-25

**Authors:** Yeswanth Gogisetti, Monika Pathania, Sunita Mittal, Pradeep Yadav, Prabin Kharibam, Ravi Kant

**Affiliations:** 1 Internal Medicine, Army Medical Corps, C/O 99APO, IND; 2 Internal Medicine, All India Institute of Medical Sciences, Rishikesh, IND; 3 Physiology, All India Institute of Medical Sciences, Rishikesh, IND; 4 Internal Medicine, Sri Siddhartha Medical College, Tumkur, IND; 5 College of Nursing, All India Institute of Medical Sciences, Rishikesh, IND

**Keywords:** choice reaction time, auditory p300, moca, mmse, hypertension

## Abstract

Background: Hypertension is an established risk factor for dementia, and the prevalence of hypertension and dementia is rising. Current tests to diagnose cognitive dysfunction at an early stage lack sensitivity and specificity. Recently event-related potentials (ERPs) have gained much attention in diagnosing cognitive dysfunction and are independent of the education status of the subject. This study was done to find any cognitive deficits in the hypertensive population with electrophysiological evidence, which might open the doors for the need to screen the population at an earlier stage so that the population can be prevented from dementia.

Methods: Some 31 middle-aged (18-65 years) hypertensives were compared with 31 age, sex, education, and handedness matched normotensives about cognition by neuropsychometric test battery including Hindi Mini-mental Status Examination (HMSE), Hindi Montreal Cognitive Assessment (MoCA), choice reaction time (CRT), and auditory event-related potentials.

Results: Hypertensives and normotensives differed significantly concerning P300 potentials’ latency (Fz and Cz P300 latencies: p-value: 0.001), and this change was correlated well with the duration of diastolic blood pressure (BP) (r-value: 0.670). The remaining tests, HMSE, Hindi MoCA, and CRT, were dependent on the education status of the patient.

Conclusions: The effect of hypertension on cognitive impairment is evident and can be proved early in its pre-clinical stage using ERPs. Early identification can help in specifying high-risk individuals. ERPs have great potential in screening and diagnosing and can also help in assessing cognition as a reliable tool to show the effect of treatments/interventions on cognitive defects.

## Introduction

Hypertension is an established risk factor for cognitive decline, and anti-hypertensives’ role in preventing the same further substantiates it [1-3]. In 2000, the world was estimated to have nearly one billion people with hypertension, increasing to 1.56 billion by 2025 [4].

The findings of cross-sectional studies of blood pressure (BP) and cognitive function have significantly varied in their results, with some studies having higher rates of cognitive impairment associated with elevated BP, others with low BP, and also U-shaped and J-shaped relationships [5-7]. However, longitudinal studies showed a positive correlation between hypertension and cognitive decline. Randomized studies showed a varied picture regarding the effects of treatment of hypertension on cognition [2]. Not only cognitive function but also hypertension is also shown to increase oxidative stress levels and decrease heart rate variability predisposing a person to an increased risk of cardiac morbidity and mortality [8]. The quality of life and sleep were also found to be poor among hypertensive patients [8-9].

After age 65, there is an exponential increase in lacunae and white matter changes [10], which might hinder the detection of early cognitive deficits due to hypertension at a stage when early intervention can prevent further deterioration, as the process of cognitive decline is irreversible. The controversy about the association between later life hypertension and cognitive decline arises because the longitudinal relationship between cognitive change and BP is sensitive to the effects of age, duration of follow-up and number of BP measurements, hypertensive treatment status, comorbidity with cardiovascular diseases and stroke, and possibly subclinical dementia [3].

Recently cognitive functions were able to assess objectively by event-related potentials (ERPs) and have become an essential tool in evaluating dementia and related diseases. The advantage of these tests is that they are independent of the patient’s education level and region. Significant functional impairment can be localized as these are recorded in different areas of the scalp [11]. Among traditional ERP components, the P300 has been most frequently explored. Occurring between 250 and 600 msec following stimulus presentation, the P300 is presumed to reflect processes related to working memory and contextual updating [12].

Vascular cognitive impairment caused by hypertension often involves executive functioning in the early stages, which cannot be well picturized in Mini-Mental Status Examination (MMSE). In comparison, Montreal Cognitive Assessment (MoCA) has a better chance of finding the same [5]. Hence a battery of tests, including MMSE (Hindi version - HMSE), MoCA (Hindi MoCA), choice reaction time (CRT), and auditory brain ERPs, were used in our study for better assessment of cognitive impairment at an early stage.

The study was carried out to look for electrophysiological evidence of cognitive deficits among the hypertensive population at an early stage. This study will enlighten the need to screen the hypertensive population for the cognitive deficit to prevent full-blown dementia.

## Materials and methods

The cross-sectional analytical study was conducted in a tertiary care hospital. Thirty-one hypertensives and 31 normotensive subjects with matched age, sex, handedness, and education were taken in the study. Patients attending out-patient department (OPD) diagnosed with hypertension [13], whose age, gender, handedness, and education matched with the normal population were included in the study. Excluded patients were those aged <18 years and >65 years, diagnosed with cognitive impairment and psychiatric illness, central nervous system illness, and chronic diseases including diabetes mellitus, chronic kidney disease, chronic liver disease, chronic obstructive pulmonary disease, and connective tissue disorders. Patients with psychiatric illness or taking psychotropic drugs, alcoholics (according to ICD 10 criteria), consumption of alcohol and caffeine within 24 h of the experimental session, and patients with hearing difficulty who report hearing difficulty on questioning “do you feel you have a hearing loss?”[14], were excluded from the study.

Patient evaluation

**Figure 1:**
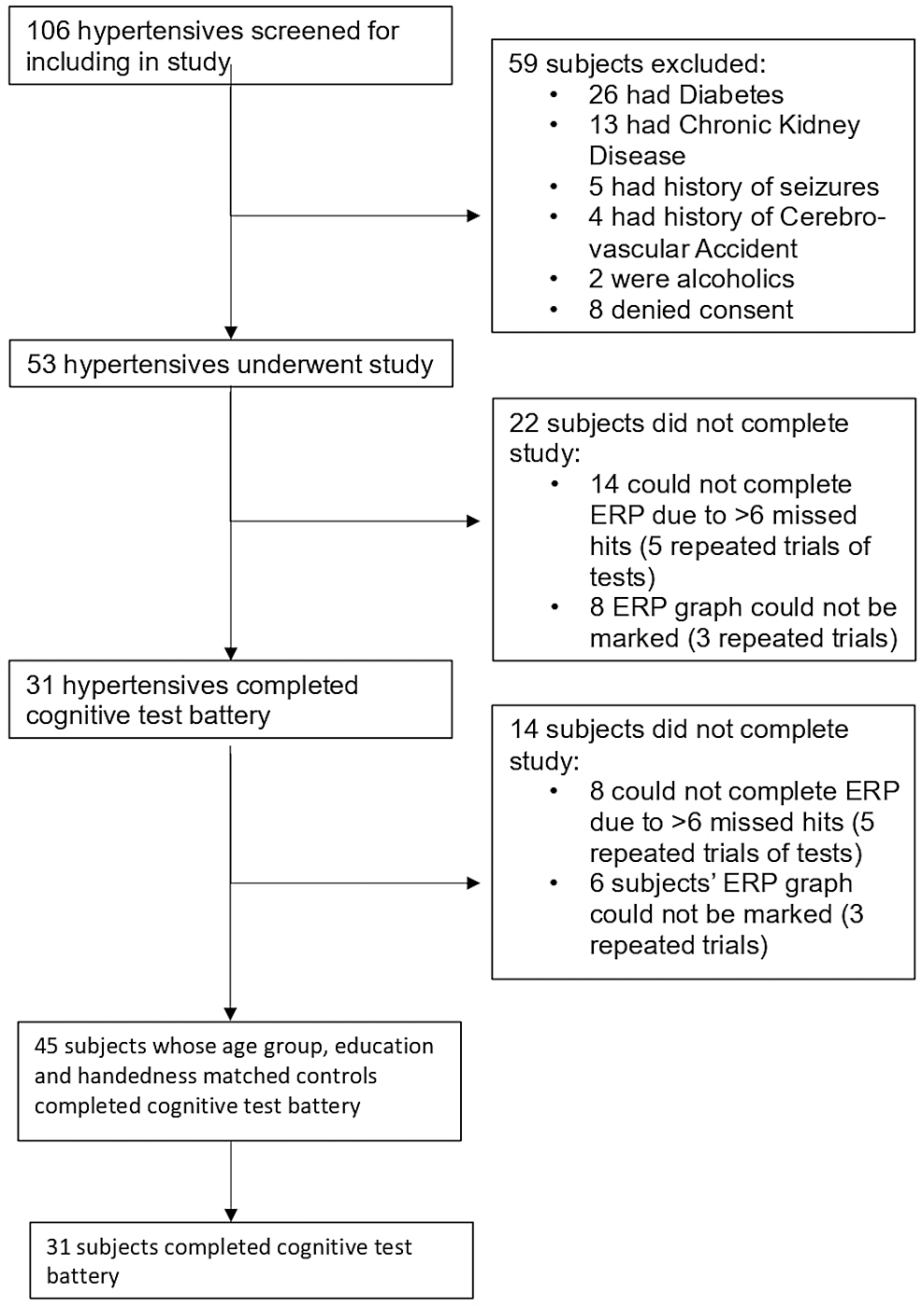
Study flow chart.

After taking informed consent, a detailed history was taken, and a physical examination was performed and recorded in a pre-developed proforma (Appendix 1). BP was measured in all subjects according to American Heart Association (AHA) guidelines [13]. The flow chart for the study is given in Figure 1.

The battery of tests for neuro-psychometric assessment [ERP-P300, Hindi Mini-Mental Status Examination (HMSE), Hindi MoCA, and choice reaction time (CRT)] were applied in that order for uniformity.

Auditory P300 

Guidelines were practised as guided by Picton et al. [15]. Subjects were informed about the procedure the day before the scheduled ERP test on a phone call and advised for a head bath on the day of testing and to abstain from caffeine and alcohol for 24 h before the test. After explaining the procedure in the laboratory, he/she was seated comfortably, the procedure was explained, and written/informed consent for doing the electrophysiological study was taken from each subject. The skin over the electrode site was cleaned with Nuprep® (Weaver and Company, Aurora, CO) gel and electrodes were attached using Konix® (Turkauz Kong, Instanbul, Turkey) paste. Pure silver disk electrodes (Nihon Kohden, Tokyo, Japan) were used. The electrodes were attached according to the standard protocol, and the test was run after giving the necessary instructions to the patient. Subjects were asked to keep their eyes open as closing eyes can lead to alpha waves in EEG, which can contaminate the response waveform. Odd-ball paradigm was applied in the test, where two types of auditory stimuli (two different frequencies - target and non-target) were given to the subject, and he/she needed to ignore the frequent (non-target) high-frequency tone and respond to the rare low-frequency tone (target) by pressing the right footpad as soon as he/she hears the target stimuli. A trial test for the first 10 stimuli was done on all subjects to familiarize themselves with the test. The detailed characteristics of P300 and its procedure are explained in Appendix 2.

HMSE and Hindi MoCA

Pre-formed validated questionnaires: HMSE [16] and Hindi MoCA [17] were asked, and scores were recorded. HMSE and MoCA used are attached in Appendices 3-4.

CRT

The CRT was recorded on already validated Deary-Liewald reaction time software on a computer with WindowsTM version 8.1 (Microsoft, Washington). This was designed by Ian J. Deary and programmed by David Liewald, with several iterations between the initial design and the final program was used here. Details of CRT are given in Appendix 5.

Statistical analysis

Signal Analysis

All the trials of P300 waves were averaged in each session. Peak amplitudes (micro-volts - μV) and latencies (milliseconds - ms) were obtained relative to a pre-stimulus baseline. Data from Cz and Fz electrodes were considered for analysis. All the values were expressed as mean ± SD. A p-value <0.05 is considered significant. All statistics were done with the help of SPSS version 21 (IBM Corp., Armonk, NY). Mean, median, and frequency percentages were calculated for descriptive statistics. An unpaired t-test is applied between hypertensives and normotensives. Similarly, the same test is applied between males and females. Analysis of variance (ANOVA) is applied between age groups and also in education groups. Chi-square is applied to see the association between gender and education. Pearson correlation is applied to see the correlation between the variables. The correlation coefficient is expressed as r-value along with p-value.

## Results

In our study, 31 hypertensive subjects were compared with 31 normotensive subjects regarding cognitive parameters. They were analyzed based on age, gender, education, BMI, and BP status, including the duration of hypertension in hypertensives, as given in Table 1.

**Table 1:** Frequencies and percentages of baseline clinical characteristics in subjects of both groups. HT, hypertensive; NT, normotensive

Variable	Subgroup	HT [N(%)]	NT [N(%)]
Age group	25-34 years	4 (12.9%)	4 (12.9%)
	35-44 years	12 (38.7%)	12 (38.7%)
	45-54 years	13 (41.9%)	13 (41.9%)
	55-64 years	2 (6.5%)	2 (6.5%)
Sex	Male	18 (58.1%)	18 (58.1%)
	Female	13 (41.9%)	13 (41.9%)
Education level	Illiterate	5 (16.1%)	5 (16.1%)
	Primary (1st–5th class)	2 (6.5%)	2 (6.5%)
	Secondary (6th-10th class)	7 (22.6%)	7 (22.6%)
	Intermediate (11th and 12th class)	6 (19.4%)	6 (19.4%)
	Degree (13- 15 years of education)	7 (22.6%)	7 (22.6%)
	Post-Graduate (>15 years of education)	4 (12.9%)	4 (12.9%)
Handedness	Right	31 (100%)	31 (100%)
	Left	0	0

The HMSE, Hindi MoCA, and CRT scores did not differ between the groups. Cz and Fz P300 latencies differed significantly between the groups, with hypertensives showing longer latencies (p value: 0.001), but their amplitudes did not differ significantly (Table 2). 

**Table 2:** The comparison of dependent variables between HT and NT. HT, hypertensive; NT, normotensive; HMSE, Hindi Mini-mental Status Examination; MoCA, Montreal Cognitive Assessment; CRT, choice reaction time

Variable	HT (31) (mean ± SD)	NT (31) (mean ± SD)	p-value
HMSE	28.71 (±2.25)	29.39 (±1.61)	0.178
Hindi MoCA	24.97 (±5.35)	26.19 (±4.36)	0.327
CRT	873.69 (±231.68) ms	843.02 (±264.26) ms	0.629
Cz P300	348.10 (±28.55) ms	319.65 (±34.93) ms	0.001
Cz N100	97.65 (±16.35) ms	96.42 (±12.27) ms	0.74
Cz N200	234.16 (±33.64) msec	224.97 (±30.99) ms	0.268
Cz P200	179.16 (±28.54) ms	173.81 (±21.57) ms	0.408
Fz P300	350.23 (±33.48) ms	313.94 (±32.29) ms	<0.001
Fz N100	100.97 (±10.75) ms	92.55 (±19.70) ms	0.041
Fz N200	240.97 (±32.02) ms	223.94 (±27.31) ms	0.028
Fz P200	171.45 (±37.68) ms	166.10 (±19.46) ms	0.485
Cz P300-N100	15.65 (±8.12) μV	16.39 (±5.75) μV	0.68
Cz N200-P200	6.26 (±4.13) μV	8.26 (±4.37) μV	0.069
Fz P300-N100	14.19 (±8.79) μV	15.61 (±5.83) μV	0.457
Fz N200-P200	7.10 (±4.96) μV	9.68 (±6.24) μV	0.076

**Figure 2:**
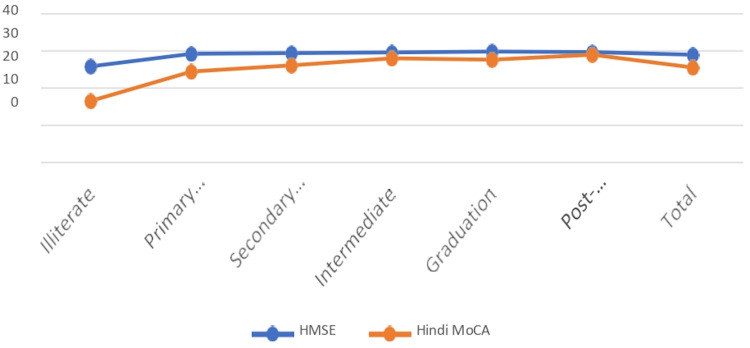
Effect of education on HMSE and Hindi MoCA. p-value: <0.001 for both HMSE and MoCA HMSE, Hindi Mini-mental Status Examination; MoCA, Montreal Cognitive Assessment

The effect of education was compared in all subjects (Table 3). HMSE and Hindi MoCA scores differed significantly between the education groups (Figure 2). The effect of education was not significant on ERP parameters (Appendix 6 -- Table 4).

**Table 3:** Comparing mean HMSE and Hindi MoCA scores in between education groups by ANOVA. ANOVA, analysis of variance; HMSE, Hindi Mini-mental Status Examination; MoCA, Montreal Cognitive Assessment; CRT, choice reaction time

Dependent variable	Education level	N	Mean	Standard deviation	p-value
HMSE	Illiterate	10	25.9	3.348	<0.001
	Primary	4	29.25	0.5	
	Secondary	14	29.5	0.855	
	Intermediate	12	29.67	0.492	
	Graduate	14	29.86	0.363	
	Post-graduate	8	29.75	0.463	
	Total	62	29.05	1.97	
Hindi MoCA	Illiterate	10	16.6	4.274	<0.001
	Primary	4	24.5	1.915	
	Secondary	14	26.14	3.11	
	Intermediate	12	28	2.089	
	Graduate	14	27.71	1.978	
	Post-graduate	8	29	1.195	
	Total	62	25.58	4.881	
CRT	Illiterate	10	1193.394	104.56789	<0.001
	Primary	4	1133.3813	166.05199	
	Secondary	14	922.6168	168.58805	
	Intermediate	12	711.6083	192.47481	
	Graduate	14	740.3846	126.87363	
	Post-graduate	8	616.1525	144.90086	
	Total	62	858.3549	246.94173	

**Figure 3:**
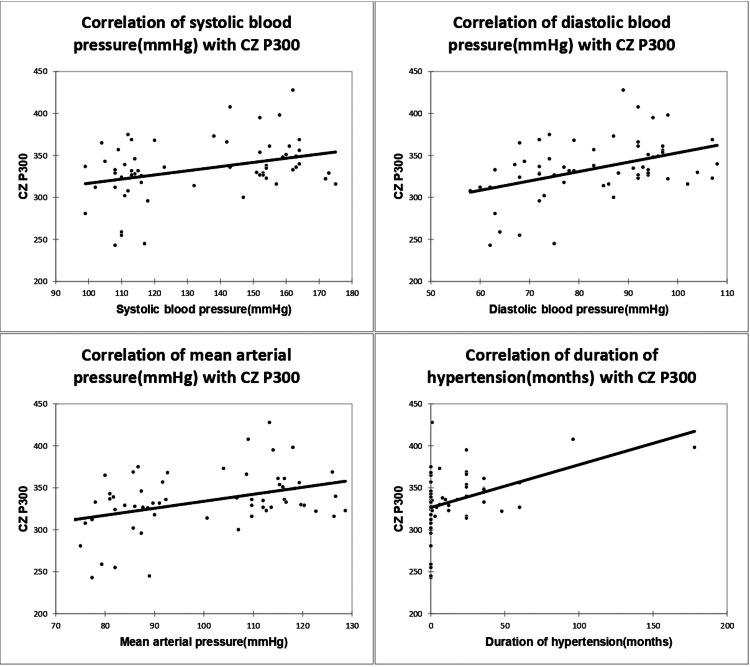
Correlation of SBP, DBP, MAP, and duration of hypertension with Cz P300, with a correlation coefficient of 0.35, 0.439, 0.405, and 0.418, respectively. SBP, systolic blood pressure; DBP, diastolic blood pressure; MAP, mean arterial pressure

**Figure 4:**
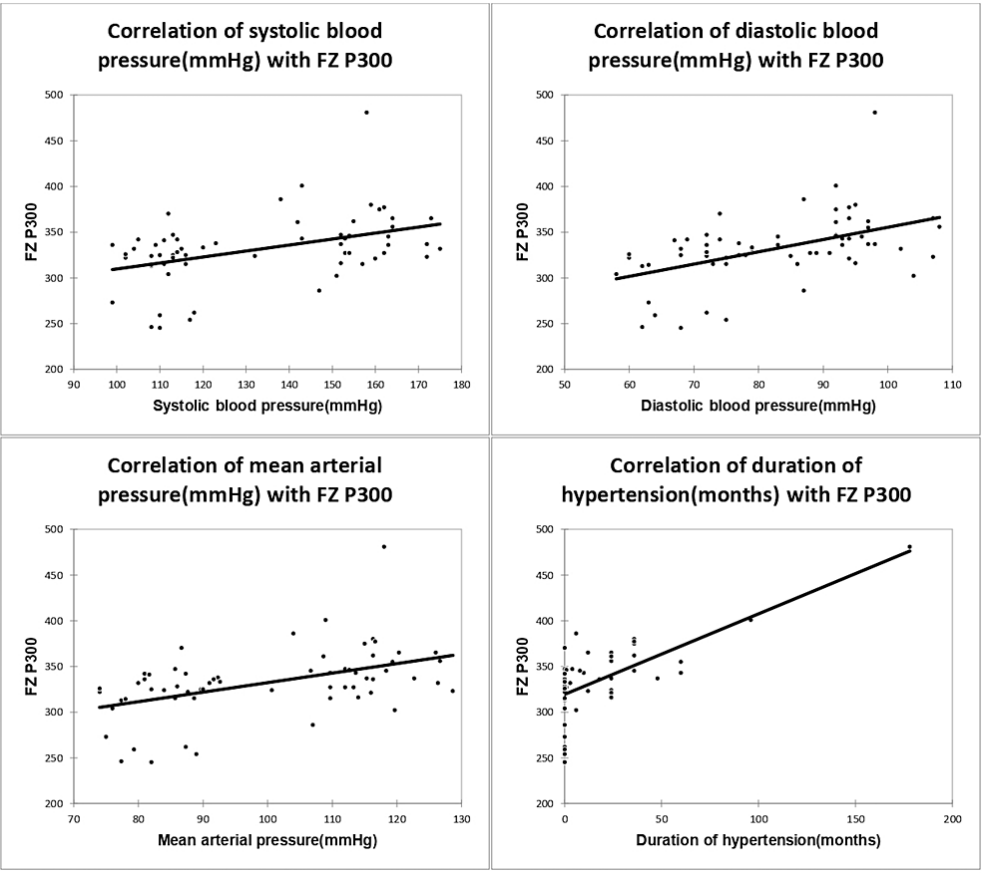
Correlation of SBP, DBP, MAP, and duration of hypertension with Fz P300, with a correlation coefficient of 0.428, 0.49, 0.47, and 0.67, respectively. SBP, systolic blood pressure; DBP, diastolic blood pressure; MAP, mean arterial pressure

Hypertension parameters including systolic BP, diastolic BP, mean arterial pressure, and hypertension duration correlated significantly with Cz P300 and Fz P300 (Table 4; Figures 3-4).

**Table 4:** Correlation of blood pressure and duration of hypertension with Cz P300 and Fz P300. SBP, systolic blood pressure; DBP, diastolic blood pressure; MAP, mean arterial pressure

Variables	SBP (mmHg)	DBP (mmHg)	MAP (mmHg)	Duration of hypertension (months)
CZ P300				
Correlation coefficient	0.35	0.439	0.405	0.418
p value	0.005	0.0004	0.001	0.001
FZ P300				
Correlation coefficient	0.428	0.49	0.47	0.67
p value	0.001	0.0001	0.0001	<0.0001

**Figure 5:**
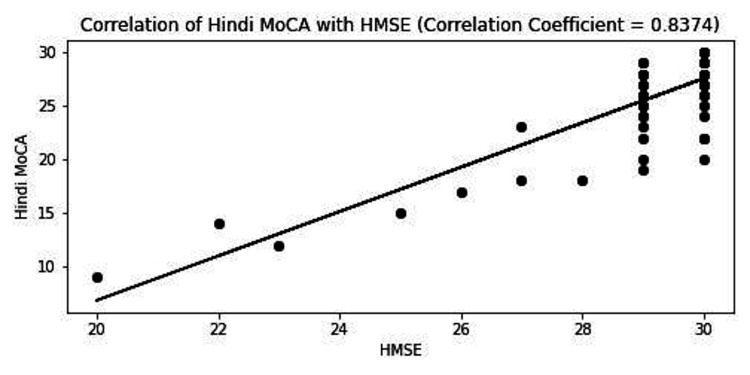
Correlation of Hindi MoCA with HMSE. MoCA, Montreal Cognitive Assessment; HMSE, Hindi Mini-mental Status Examination

**Figure 6:**
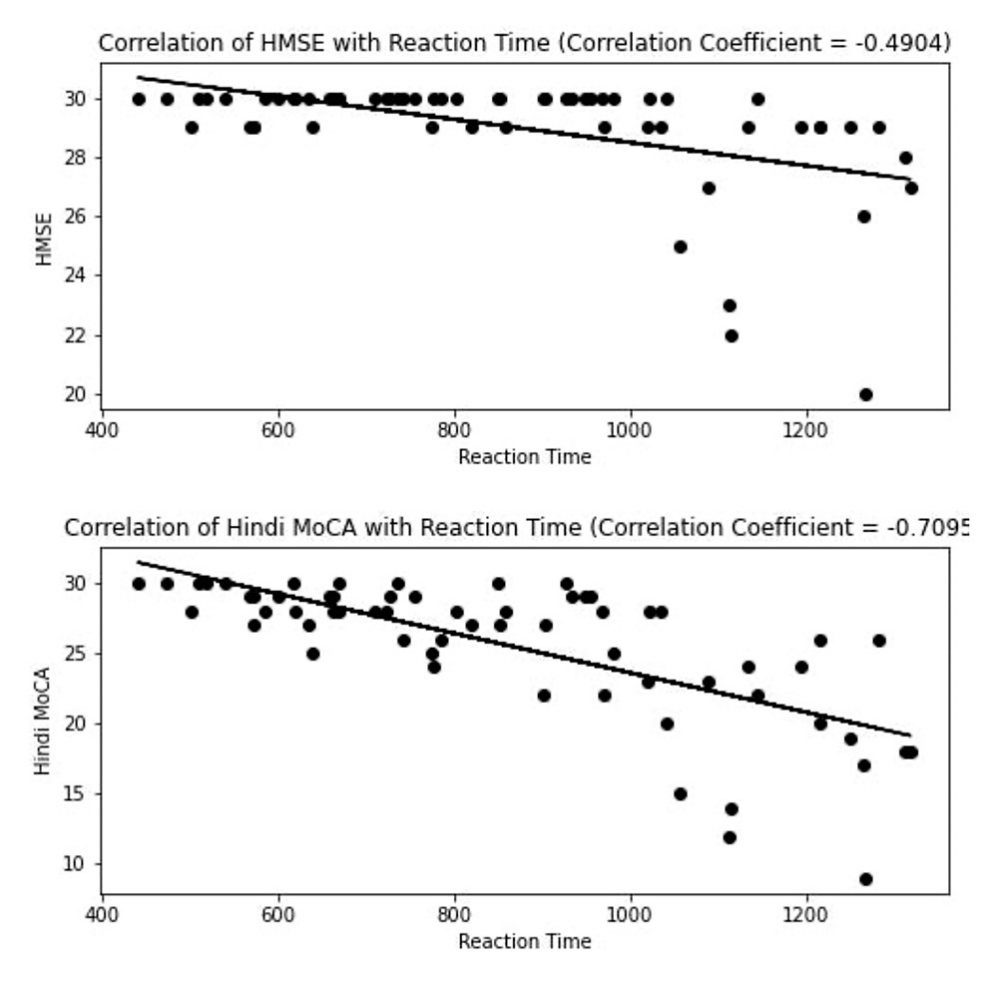
Correlation of HMSE and Hindi MoCA with CRT. The correlation coefficient of HMSE and CRT are -0.49 and -0.709 for Hindi MoCA with CRT. HMSE, Hindi Mini-mental Status Examination; CRT, choice reaction time; MoCA, Montreal Cognitive Assessment

HMSE and Hindi MoCA correlated well among themselves, with a positive correlation of r=0.837 (p-value < 0.001) (Figure 5). They also negatively correlated with reaction times (p-value < 0.001), indicating their common dependence on education (Figure 6).

## Discussion

The implication of hypertension on the cognitive decline has varied results in the previous studies [5-7]. However, all the studies that used ERPs to evaluate cognitive dysfunction proved its effect with electrophysiological evidence [18-21]. 

Hypertensives and normotensives did not significantly differ regarding demographic variables in our study. Age, sex, education, and handedness were matched to remove their confounding effect on dependent variables (cognitive function parameters). BMI was significantly higher in the hypertensive group. Except for the BMI and hypertension status, other independent variables (including age, age group, sex, and education level) did not significantly differ between the two groups. 

The mean age group of the individuals was 43.29 (±7.84) years in hypertensives and 41.81 (±7.76) years in normotensives. Some 80.6% of individuals were of age 35-54 years. This age group was considered for study since age >65 years itself has more implications on cognitive decline.

Hypertensives and normotensives significantly differed in Cz P300, Fz P300, Fz N100, and Fz N200 latencies. No other parameters, including HMSE scores, Hindi MoCA scores, reaction times in CRT including correct reaction time, reaction time, or wrong attempts, did not differ significantly between the groups. This shows the effect of hypertension on cognition which is represented here by the prolongation of ERP wave latencies. This effect is not evident in HMSE, Hindi MoCA, and CRTs. The increase in P300 latencies is well documented in previous studies [18-21], and also increase in N200 latencies is shown in studies done in the past by Si et al. and Akhtar et al. [22-23]. 

Mini-Mental Status Examination has a sensitivity of 18% to detect mild cognitive impairment. Though MoCA has higher sensitivity of 100%, its specificity falls to 82% and has a chance of false positivity. Though MoCA is found to be a reliable screening tool for cognitive impairment, its validity in low-income countries is still questionable, where the literacy rate is low [24]. HMSE and Hindi MoCA are significantly associated with a patient’s education level and are not sensitive to recognizing early cognitive deficits. These cognitive deficits are expected to be not very evident in the early years of hypertension to be picked up in HMSE and Hindi MoCA [17]. 

Several studies done previously interpreted similar results [18-21]. Few showed a significant decrease in amplitude, but our study did not reproduce such results. The advantage of easy reproducibility and reliability can make this ERP test a standard test to pick up early cognitive deficits [25]. Moreover, it is a non-invasive test that can favor patients’ compliance for follow-up studies. The results did not show conflictive evidence compared to previous studies [18-21]. The association of hypertension with cognitive deficits, though, lacks unanimous conclusions as low BP also proved to be a risk factor for the same [26], but all the studies done with the help of ERP showed a positive association of hypertension with cognitive deficits.

Individuals with all levels of education status, including illiterates to post-graduates, were included in the study. As matching was done, there was no difference between the groups. HMSE and Hindi MoCA were significantly associated with the association of education with dependent variables. There was a strong positive correlation between these scores with education. The association of education with these tests has already been a fact [27]. A similar association was found with CRT, primarily due to the usage of computers. 

Hypertension significantly affects cognition, which can be picked up early with the assistance of ERP. The use of MMSE and MoCA scales is primarily dependent on the education level of the subject. Though Hindi versions of both the tests were used in the study, they could not help in detecting early cognitive deficits by eliminating the confounding effect of education. Nevertheless, the use of ERP in detecting cognitive deficits that very early seem to be promising in the near future. Similar conclusions are made by Papaliagkas et al. in a similar study [28]. The effect of hypertension treatment on cognition has also been recently studied, and we have supportive data that treatment of hypertension has decreased the risk of cognitive impairment [29]. Though the treatment of cognitive deficits that are already established is not well known, identifying the risk factors and preventing the dreaded complication is a very effective way of dealing with it and a cost-effective solution. The future is promising regarding the development of tools with the help of artificial intelligence to fight cognitive impairment and reverse at least to some extent though it is in its nascent stage right now [30]. 

Limitations

 The additional use of the Pz electrode for studying P300 would have helped in better understanding, and generalized results would have conveyed the reproducibility of findings in all the electrodes. A larger sample size would definitely increase the power of the study, as the prevalence of hypertension in the population is high. A follow-up study would firmly establish the association and causation of hypertension in cognitive impairment. 

## Conclusions

The effect of hypertension on cognitive impairment is evident and can be objectively proved early in its pre-clinical stage using event-related potentials. Screening for pre-clinical cognitive deficits can significantly change cognitive disorders' course. Early identification can help specify high-risk individuals and thus can either help us alter modifiable risk factors or guide us in deciding on any intervention for treatment or prevention. As ERPs alone have the potential in the current era to catch very early deficits, they can be considered a game changer in dealing with cognitive disorders. They are beneficial in screening and diagnosing and can help assess cognition as a reliable tool to show the effect of treatments/interventions on cognitive defects. Clinical use of ERPs, which are now being used chiefly as experimental tools, must be revisited, encouraged, and inculcated in practice to prevent cognitive dysfunctions in hypertensive subjects. Early screening and a multimodal non-pharmacological approach focusing on good sleep quality, assessing heart rate variability, and screening for cognitive deficit should be adopted in treating hypertension to prevent the neglected long-term complications of hypertension for a good quality of life.

## Figures and Tables

**Figure 7:**
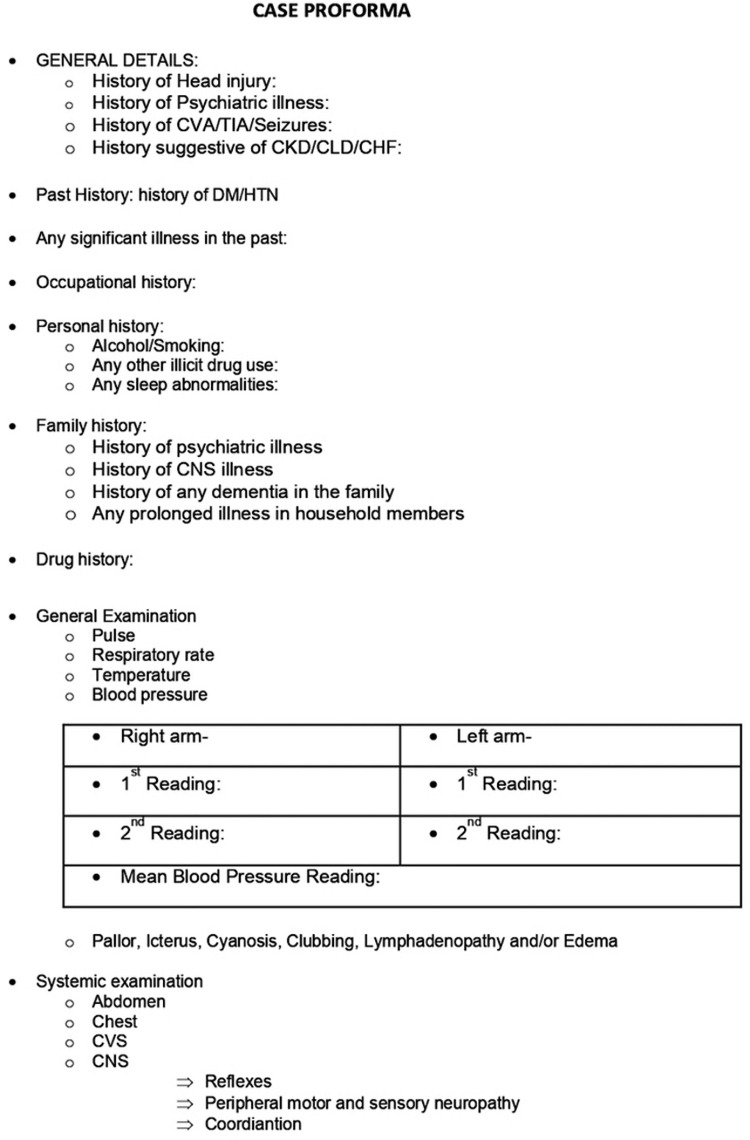
Case proforma used in initial evaluation of subjects.

**Figure 8:**
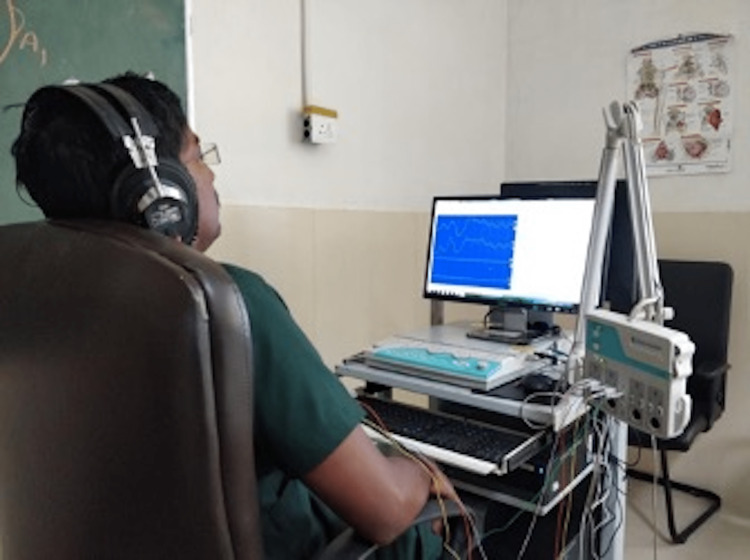
Subject performing auditory P300 test.

**Figure 9:**
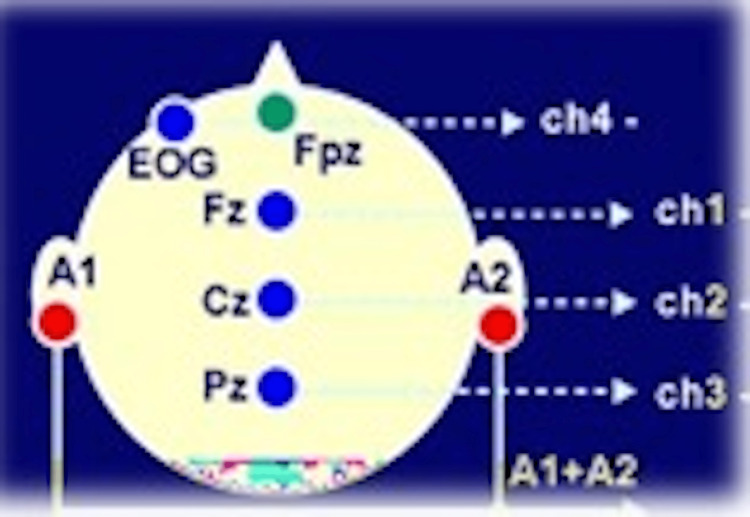
Electrode placement site over the scalp for auditory P300 test.

**Figure 10:**
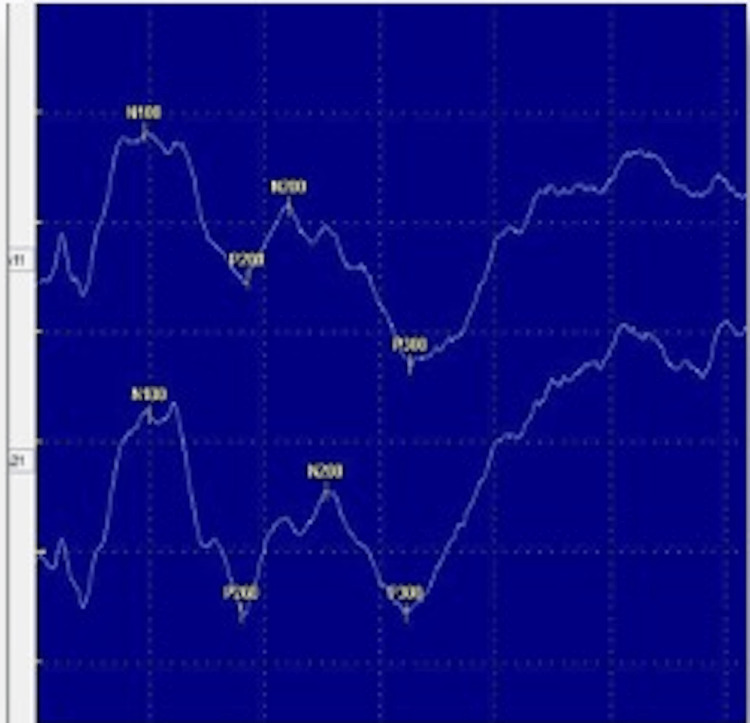
Sample ERP graph with marking of potentials. ERP, event-related potential

**Figure 11:**
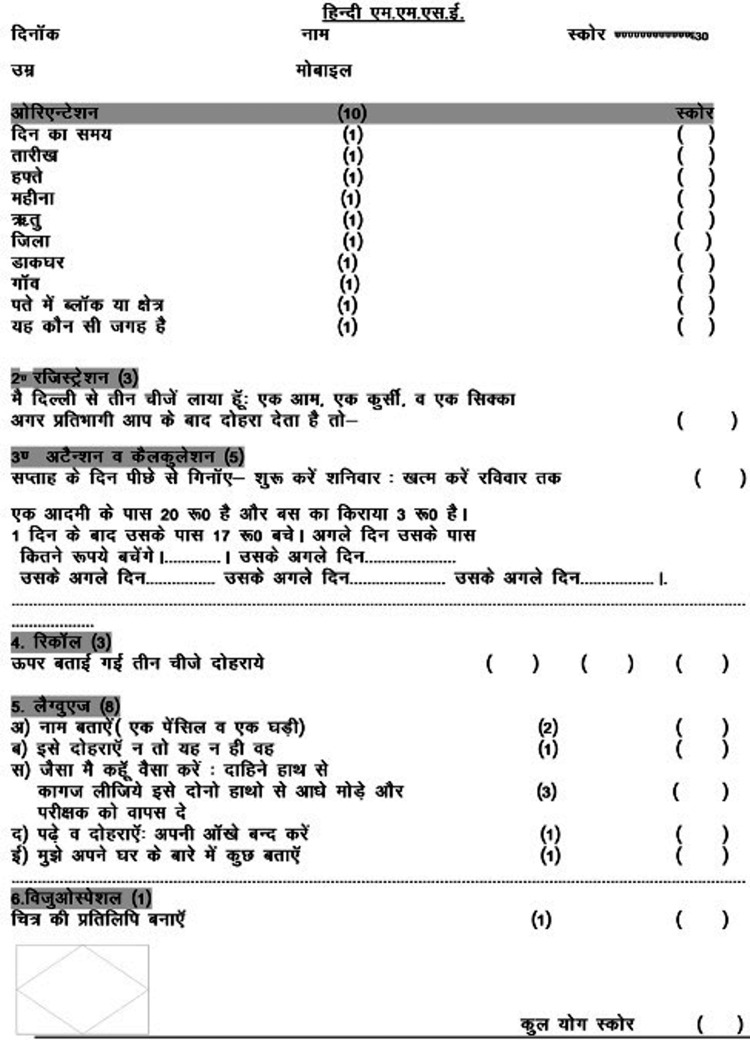
Hindi MMSE. MMSE, Mini-Mental Status Examination

**Figure 12:**
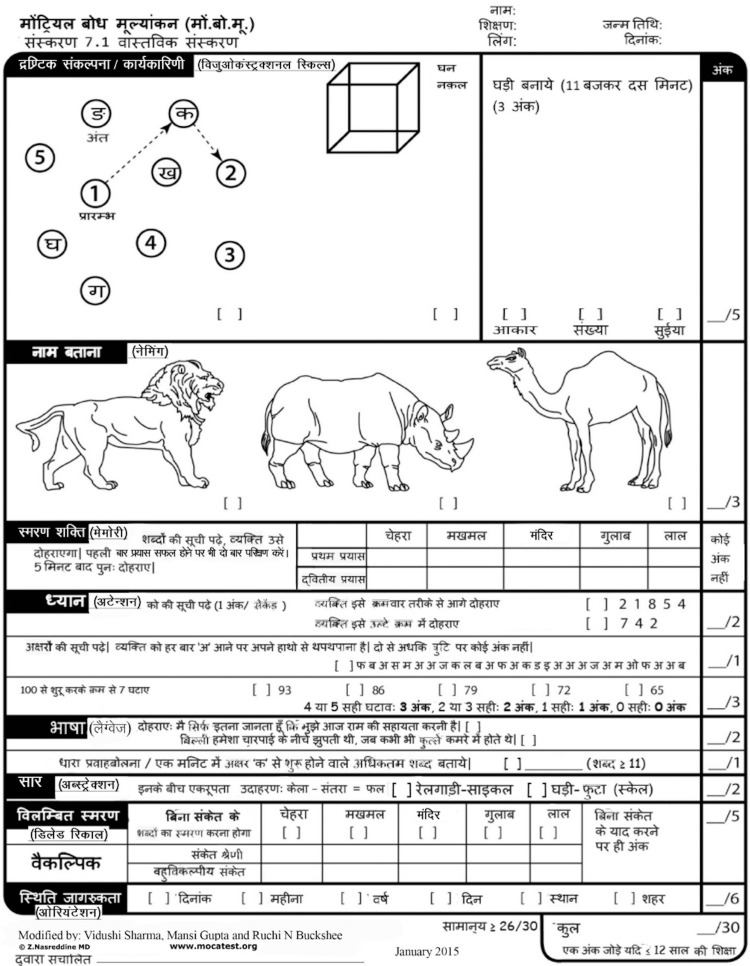
Hindi MoCA. MoCA, Montreal Cognitive Assessment

**Figure 13:**
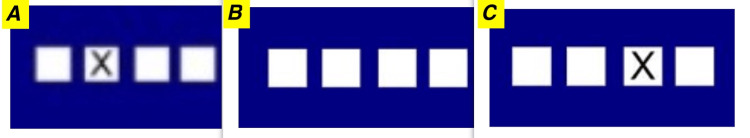
Sample of CRT screenshot. A - sample CRT screenshot where ‘x’ or ‘2’ to be pressed; B - inter-stimulus screen in CRT; C - sample CRT screenshot where ‘,’ or ‘3’ to be pressed CRT, choice reaction time

## Appendices

Appendix 1

Please see Figure 7 below.

Appendix 2

Auditory P300

Guidelines were practised as guided by Picton. Subjects were informed about the procedure the day before the scheduled ERP test on a phone call and advised for a head bath on the day of testing and to abstain from caffeine and alcohol for 24 h before the test. They were allowed to take their routine medication (like anti-hypertensives in subjects who are already on them). Newly diagnosed cases of hypertension and those hypertensives who are not started on medication till the test are advised to start medication as advised after the test.

After explaining the procedure in the lab (Electrophysiology lab) he/she was seated comfortably, the procedure was explained and informed consent for doing the electrophysiological study was taken from each subject (Figure 8).

All subjects were asked to relax for 5 minutes in the lab. No one else except the investigator, patient and patient’s attendant was allowed in the lab during the study. A security guard is placed outside the lab and instructed to ensure the same. After explaining the procedure, the silence was maintained except for words of encouragement given by the instructor during the test infrequently. Cell phones of all three members in the room were either switched off or kept in silent mode during the procedure. The skin over the electrode site was cleaned with Nuprep® gel and electrodes were attached using Konix® paste in the electrode placement site (Figure 9).

Pure silver disk electrodes (Nihon Kohden) were used. The electrodes were attached according to the standard protocol and the test was run after giving the necessary instructions to the patient. Skin-electrode impedance was reduced to 5KW or less. Subjects were asked to keep their eyes open as the closing of eyes can lead to alpha waves in EEG which can contaminate the response waveform. Odd-ball paradigm was applied in the test, where two types of auditory stimuli (two different  frequencies→ targets and non-target)  were given to the subject and he/she needs to ignore the frequent (non-target) high-frequency tone and respond to the rare low-frequency tone (target) by pressing the right footpad as soon as he/she hears the target stimuli. The frequency and probability of the stimuli are described below. The timing and probability of the stimuli were automated in the software. A trial of test for the first 10 stimuli was done on all subjects for familiarizing with the test. A repeat test was also done in a few subjects in view of obtaining better electrophysiological graphs, previously which were having difficulty with marking and interpreting amplitudes and latencies of N100, N200, P200, and P300. A sample ERP is given in Figure 10.

Characteristics in P300

·      Tones: two categories- one rare (target) and one frequent (non-target)

·      Frequency: 1000 Hz (non-target) and 2000 Hz (target)

·      Intensity: 40 dB

·      Tone burst rise: 10 ms

·      Plateau: 100 ms

·      Sensitivity: 20-50 uV/div

·      Analysis time: 100 ms/div

·      Trigger delay: -1 div

·      Probability: 80% non-target, 20% target

·      Filter: Hi- 50-100 Hz, Low-0.1 Hz

Participant

§  Position: seated with both arms and back resting

§  Eyes: open

§  Active task: participant needs to press the button by right foot at foot pad on hearing each target stimulus

§  Minimal configuration: Fz, Cz (active negative electrodes channel 1 and channel 2 respectively).

§  Reference: electrode on one ear lobe as an online reference and other on the opposite ear for offline reference (A1 and A2 positive electrodes).

§  Ground: Fpz

 Marking

•              N100: negative peak at around 100 ms

•              N200: next negative peak between 150 ms and 300 ms

•              P200: positive peak usually between N100 and N200 and before P300 between 100 m and 300 ms

•              P300: positive peak after 200 ms-500 ms after P200

•              Amplitude: voltage difference between N100 and P300, N200 and P200 were measured.

Response times were generated depending on the time taken in milliseconds from stimulus to motor response that is from hearing the target stimulus to pressing the right foot pad. Mean response time, and minimum and maximum response times were given automatically by the software in the results. Missed hits were calculated by the number of missed motor responses.

Rejection criteria for waveforms

•              Waveforms with no markable positive or negative peaks and with no significant amplitude (0 uV) for up to three trials

•              High background noise

•              >6 missed hits for up to five trials

Appendix 3

Please see Figure 11 below.

Appendix 4

Please see Figure 12 below.

Appendix 5 

Choice reaction time

The Choice reaction time was recorded on already validated Deary - Liewald reaction time software on a computer with WindowsTM version 8.1. This was designed by Ian J. Deary and programmed by David Liewald, with several iterations between the initial design and the final program that was used here.

All the testing was carried out at a fixed time in the morning (9 am-12 pm) to avoid the confounding effect of the circadian rhythm. Four white squares appear in a horizontal line across approximately the middle of the computer screen, set against a blue background. Four keys on a standard computer keyboard correspond to the different squares. The position of the keys corresponds in alignment to the position of the squares on the screen: the ‘z’ key corresponds to the square on the far left, the ‘x’ key to the square second from the left, and the ‘comma’ key to the square second from the right and the ‘full-stop’ key to the square on the far right. The stimuli to respond appear in a diagonal cross within one of the squares. Participants are instructed to gently rest the index and middle fingers of their left hand on the ‘z’ and the ‘x’ keys, and the index and middle fingers of their right hand on the ‘comma’ and ‘full stop’ keys. A cross appears randomly in one of the squares and participants are asked to respond as quickly as possible by pressing the corresponding key on the keyboard. Each cross remains on the screen until one of the four keys is pressed, after which it disappears and another cross appears shortly after. The inter-stimulus intervals range between 1 and 3 seconds and are randomized within these boundaries. The computer program records the response times for each cross, the inter-stimulus interval for each trial, which key was pressed and, in the case of four-choice reaction time, whether the response was correct or wrong, a sample of CRT response is given in Figure 13.

For the ease of illiterates and subjects not accustomed to computers, ‘1’, ‘2’, ‘3’, ‘4’ keys, were used, instead of ‘z’, ‘x’, ‘,’(comma), ‘.’(full-stop) keys. The same is taken care of for both hypertensives and controls. The reaction times including correct response time, wrong response time, the number of wrong attempts and inter-stimulus interval were automatically saved in excel format by the software.

Appendix 6

Please see Table 5 below.

**Table 5:** Auditory ERPs among the various education levels. Latencies, potentials, and response time of the ERP waves of both Cz and Fz electrode were compared between the education levels by ANOVA followed by post-hoc Bonferrini tests. In latencies only Fz N200 showed significant difference between the education groups with lower latency periods in higher education categories and longer latencies in lower education groups. But this test when subjected to Bonferrini test did not yield any statistically different results in between the groups. No statistically significant difference was found in the groups between HTs and NTs with regard to potentials. Similar to reaction time derived in CRT, response time showed statistically significant difference between the education groups including mean response time and maximum response time, although minimum response time did not show statistically significant different results. All these parameters were slower in less educated subjects. On applying Bonferrini test, the significant difference was found between illiterates and post-graduates in mean and maximum response time, with higher response times in illiterates compared to post-graduate. ERP, event-related potential; CRT, choice reaction time; HT, hypertensive; NT, normotensive

Auditory ERPs					
Dependent variable	Education level	N	Mean	Standard deviation	p-value
Latencies					
Cz P300	Illiterate	10	340.7	45.181	0.452
	Primary	4	323	44.144	
	Secondary	14	341.07	28.84	
	Intermediate	12	342.08	27.57	
	Graduate	14	328.86	35.714	
	Post-graduate	8	314.63	33.445	
	Total	62	333.87	34.734	
Cz N100	Illiterate	10	100.9	15.118	0.475
	Primary	4	91	11.165	
	Secondary	14	102	19.838	
	Intermediate	12	94.25	14.104	
	Graduate	14	96.36	10.987	
	Post-graduate	8	91.88	6.556	
	Total	62	97.03	14.348	
Cz N200	Illiterate	10	237.7	38.922	0.166
	Primary	4	224.5	16.01	
	Secondary	14	235.07	29.479	
	Intermediate	12	231.42	43.141	
	Graduate	14	234.5	23.481	
	Post-graduate	8	200.88	19.157	
	Total	62	229.56	32.408	
Cz P200	Illiterate	10	173.2	19.719	0.126
	Primary	4	171.5	8.813	
	Secondary	14	171.57	19.91	
	Intermediate	12	192.67	32.225	
	Graduate	14	179.36	28.87	
	Post-graduate	8	162.38	18.134	
	Total	62	176.48	25.234	
Fz P300	Illiterate	10	329.6	28.776	0.539
	Primary	4	329.5	52.284	
	Secondary	14	342.71	45.784	
	Intermediate	12	342.08	33.792	
	Graduate	14	325	35.336	
	Post-graduate	8	315.25	33.38	
	Total	62	332.08	37.403	
Fz N100	Illiterate	10	102.2	15.091	0.179
	Primary	4	95.25	6.801	
	Secondary	14	101.71	10.469	
	Intermediate	12	97.67	12.716	
	Graduate	14	94.86	9.38	
	Post-graduate	8	84	33.076	
	Total	62	96.76	16.299	
Fz N200	Illiterate	10	247.1	29.976	0.029
	Primary	4	234.25	20.205	
	Secondary	14	239.64	37.586	
	Intermediate	12	240.75	23.054	
	Graduate	14	223.29	18.062	
	Post-graduate	8	204.25	35.099	
	Total	62	232.45	30.738	
Fz P200	Illiterate	10	169.2	13.959	0.092
	Primary	4	169.25	20.156	
	Secondary	14	155.21	42.883	
	Intermediate	12	186.08	24.92	
	Graduate	14	175	18.431	
	Post-graduate	8	154.88	33.825	
	Total	62	168.77	29.862	
Potentials					
Cz P300- N100	Illiterate	10	14.8	3.36	0.071
	Primary	4	22	5.715	
	Secondary	14	14.14	6.188	
	Intermediate	12	13.67	3.42	
	Graduate	14	16.14	10.406	
	Post-graduate	8	21.13	6.128	
	Total	62	16.02	6.988	
Cz N200- P200	Illiterate	10	7.3	5.272	0.609
	Primary	4	8.5	3	
	Secondary	14	7.79	4.423	
	Intermediate	12	6.75	2.989	
	Graduate	14	8.14	5.461	
	Post-graduate	8	4.88	2.949	
	Total	62	7.26	4.338	
Fz P300- N100	Illiterate	10	13.8	3.645	0.195
	Primary	4	20.5	7.853	
	Secondary	14	12.14	5.908	
	Intermediate	12	13.5	5.584	
	Graduate	14	15.79	10.312	
	Post-graduate	8	18.88	8.271	
	Total	62	14.9	7.43	
Fz N200- P200	Illiterate	10	8.8	4.984	0.794
	Primary	4	10	2.944	
	Secondary	14	9	5.698	
	Intermediate	12	6.33	4.979	
	Graduate	14	8.07	6.889	
	Post-graduate	8	9.63	7.23	
	Total	62	8.39	5.738	
Response time					
Mean	Illiterate	10	678.4	205.986	0.027
	Primary	4	542.25	77.237	
	Secondary	14	486	156.172	
	Intermediate	12	575.42	110.602	
	Graduate	14	535.43	173.088	
	Post- graduate	8	448.75	63.738	
	Total	62	544.32	161.067	
Minimum	Illiterate	10	264	100.687	0.177
	Primary	4	317.5	49.917	
	Secondary	14	305	50.192	
	Intermediate	12	342.5	79.673	
	Graduate	14	323.57	65.116	
	Post-graduate	8	298.75	38.707	
	Total	62	309.84	70.699	
Maximum	Illiterate	10	2409	1022.312	0.004
	Primary	4	1360	540.802	
	Secondary	14	1530	908.329	
	Intermediate	12	1309.17	595.337	
	Graduate	14	1471.43	761.424	
	Post- graduate	8	920	148.997	
	Total	62	1526.13	849.797	
Missed hits	Illiterate	10	3.4	1.578	0.241
	Primary	4	2.25	2.062	
	Secondary	14	1.64	1.692	
	Intermediate	12	2.08	1.564	
	Graduate	14	2.14	1.748	
	Post-graduate	8	2	1.512	
	Total	62	2.21	1.69	
